# Perceptions of co-teaching as a pedagogical approach to integrate basic and clinical sciences

**DOI:** 10.3389/fmed.2024.1383975

**Published:** 2024-07-18

**Authors:** Ivan Rodríguez-Martín, Emilia Condés, Judit Sánchez-Gómez, Clara Azpeleta, Antonio S. Tutor, Marta Lesmes, Beatriz Gal

**Affiliations:** ^1^Faculty of Experimental Sciences, Universidad Francisco de Vitoria, Pozuelo de Alarcón, Spain; ^2^Facultad de Ciencias Biomédicas y de la Salud, Departamento de Medicina, Universidad Europea de Madrid, Villaviciosa de Odón, Spain; ^3^Facultad de Ciencias Sociales y de la Comunicación, Departamento de Educación, Universidad Europea de Madrid, Villaviciosa de Odón, Spain; ^4^Facultad de Medicina, Departamento de Ciencias Médicas Básicas, Universidad San Pablo-CEU, Madrid, Spain

**Keywords:** supportive co-teaching, TBL, basic and clinical science integration, active methodologies, medicine students

## Abstract

**Background:**

Medical curricula must provide students with basic and clinical competencies for critical reasoning and diagnosing. These competencies are better acquired when basic and clinical science are taught in an integrated and collaborative manner. In this study, we investigate whether supportive co-teaching (SCT) is an effective approach to promote integrated and reasoned learning as well as to help medical students applying theoretical concepts to clinical scenarios taught in a team-based learning (TBL) framework.

**Methods:**

We conducted a concurrent mixed methods study. For the qualitative part, we performed a focus group and semi-structured interviews to clinical and basic science teachers and medical students. Using conventional content analysis, themes were identified deductively. For the quantitative part, an analytical and descriptive observational study of the 2019–2020 cohort of first-year undergraduate medical students was conducted (107 students out of 220 completed the survey). For the descriptive study, questions were grouped into 5 categories.

**Results:**

Deductive themes from the analysis include relationship between clinical and basic science teachers, knowledge integration, methodology, teamwork and integrated Medicine and curricular design. Basic science and clinical teachers highlighted their relationship as critical to increase their mutual knowledge. This was supported by the student’s opinion who very much valued their joint feedback. Regarding knowledge integration, both teachers and students found that horizontal and vertical integration enhanced applicability of basic knowledge to future clinical practice. The TBL methodology was very well perceived by both students and teachers and was highly motivating for students even though the need for commitment. Students considered that this program presented a great opportunity and expressed their interest in maintaining it in the future. These results were supported by the quantitative data.

**Conclusion:**

Our work supports the value of co-teaching in basic and clinical sciences within a TBL framework set in real clinical case scenarios. By employing this approach, students can actively apply their theoretical knowledge to clinical practice, enhancing their critical thinking, problem-solving, and clinical reasoning skills. Our findings can inform curriculum design and improved educational practice, leading to enhanced learning experiences for healthcare students and ultimately better patient care.

## Background

Active learning methodologies in Medical Education provide a powerful platform for medical students to gain a deeper understanding of clinical concepts and to develop the necessary skills to succeed in the healthcare field (1–5). Active learning approaches are often embedded into the educational theory of constructivism, which considers that: (a) significant learning occurs when we integrate new knowledge into the already established schemes; (b) learning is social and results from interaction with others, and (c) tasks that simulate the real professional situation are facilitators for learning (6). Teaching methodologies which are aligned with constructivist principles are particularly suited for developing diagnostic and clinical competences. When treating a patient, the physician must analyse the elements of the medical history and to establish a hypothesis based on prior knowledge (7). Additionally, in case report sessions, specialists interact to share knowledge in service for diagnosis and treatment (7). Developing competencies in these two areas of medical practice requires students to actively learn integrating knowledge from different perspectives (8). Moreover, face-to-face interaction enables participants to construct, monitor, and to build shared knowledge (9, 10).

Under this view, having basic scientists and clinicians working together seems suitable to communicate the importance of both disciplines and to demonstrate the scientific underpinnings of medicine and their role in clinical reasoning (11). One very straightforward approach to meeting this goal is supportive co-teaching (SCT). SCT is an effective teaching approach by which the knowledge and expertise of two or more teachers are employed to promote learning (12). SCT is time-consuming, as it requires a great deal of collaborative planning by instructors (13, 14). This may explain its reduced use in higher education, despite its introduction in the 1960s. More recently, reports of SCT have re-emerged at the undergraduate college level (11, 15). In general, students showed positive views of this approach, and their grades indicated they learned better than expected (16).

SCT within a Team Based Learning (TBL) experience has been used as a strategy for inter-professional collaboration (17). In this study, Rider and Brashers in 2006, observed that participants uniformly agreed in their support of this interdisciplinary team-learning model as an effective way to learn important skills for interprofessional collaboration. In a Pharmacy doctorate curriculum, co-teaching has been described to promote the integration of basic (pathophysiology, pharmacology, and medicinal chemistry) and clinical sciences (18). Students’ evaluation indicated that as compared to solo-teaching, during team-based co-teaching the discussion amongst peers is enhanced and the different instructors’ points of view are encouraged in a positive way (18). Interaction between students and instructors were highly beneficial to learning.

SCT applied to undergraduate medical education appears to be mostly used in small groups of learners during TBL sessions (18, 19). These studies report students mostly agreed that SCT enhanced their ability to apply basic science to clinically relevant problems. In undergraduate Medical Education, we have found few studies describing interactive co-teaching by pairing of a basic scientist and a clinician in an active large group or lecture format. Moreover, to the best of our knowledge there is no major study that report the impact of co-teaching on the student academic outcomes.

Here, we describe the use of interactive co-teaching in early years of a Medicine integrated program using a set combined methodologies: i.e., 2 h of autonomous work; 2 h of individual and team Readiness Assurance Tests (iRAT and tRAT, respectively), as well as team Application Problem (tApp) from the TBL methodology, and finally 2 h to work transversal competencies whereby students were provided real-clinical cases to trigger active discussions between them and instructors. The purpose of our study was threefold: (1) to describe a SCT-based program designed and taught by basic sciences and clinical faculty teachers within related disciplines, (2) to determine whether students and teachers perceive co-teaching as an effective pedagogical approach to integrate basic and clinical sciences, and finally, (3) to qualitatively assess whether studying basic sciences integrated on real clinical case scenarios with SCT helps students apply theoretical concepts to their clinical application.

## Methods

### Design

In this study we propose a concurrent mixed methods study in which the quantitative and qualitative research approaches are conducted simultaneously and concurrently (20). Our goals are to analyze the students and teachers’ perception of co-teaching to integrate basic and clinical sciences, and to assess whether running real clinical cases SCT under the TBL methodology can help students to translate theoretical contents into its clinical application.

### Setting

The Integrated Medicine (IM) pilot program was tested with first year medical students at the Universidad Europea of Madrid. The IM program is based on 4 real clinical cases which were developed over three consecutive sessions (2 h per session) along 12 weeks. Approximately 40 students per session worked in pre-defined groups of 5–6 students each. Group members remained constant throughout the IM program to facilitate the development of positive team dynamics.

A Curriculum Integrated Reform Group (CIRG) developed the IM program. The CIRG was involved in preparing and supervising the whole program from the beginning to the end of it. Many feedback sessions were scheduled to revise how was the IM program developing. The CIRG tasks covered the definition of the learning outcomes for each of the clinical cases, the choice of the best clinical case, the elaboration of the didactic material for the students and the preparation of each of the phases of the TBL including the evaluation part. This work was carried out during the previous academic year and involved an extra workload to ensure that the sessions within the program were as well prepared as possible. This group was formed by basic and clinical science experts. Convergent learning outcomes from the different subjects included in the IM program (Biochemistry, Cellular Biology, Physiology, Genetics, Histology, and Anatomy) were defined. The IM program follows the TBL -adapted methodology (21) which is only applied in this new IM pilot program to the first year medical students. The rest of the regulated subjects were taught following a more traditional methodology. For this purpose, a guided autonomous work session was run before the iRAT, tRAT and tApp sessions. In the final session, two transversal competencies (teamwork and the ability to apply the contents to practice) were evaluated.

**Figure 1:**
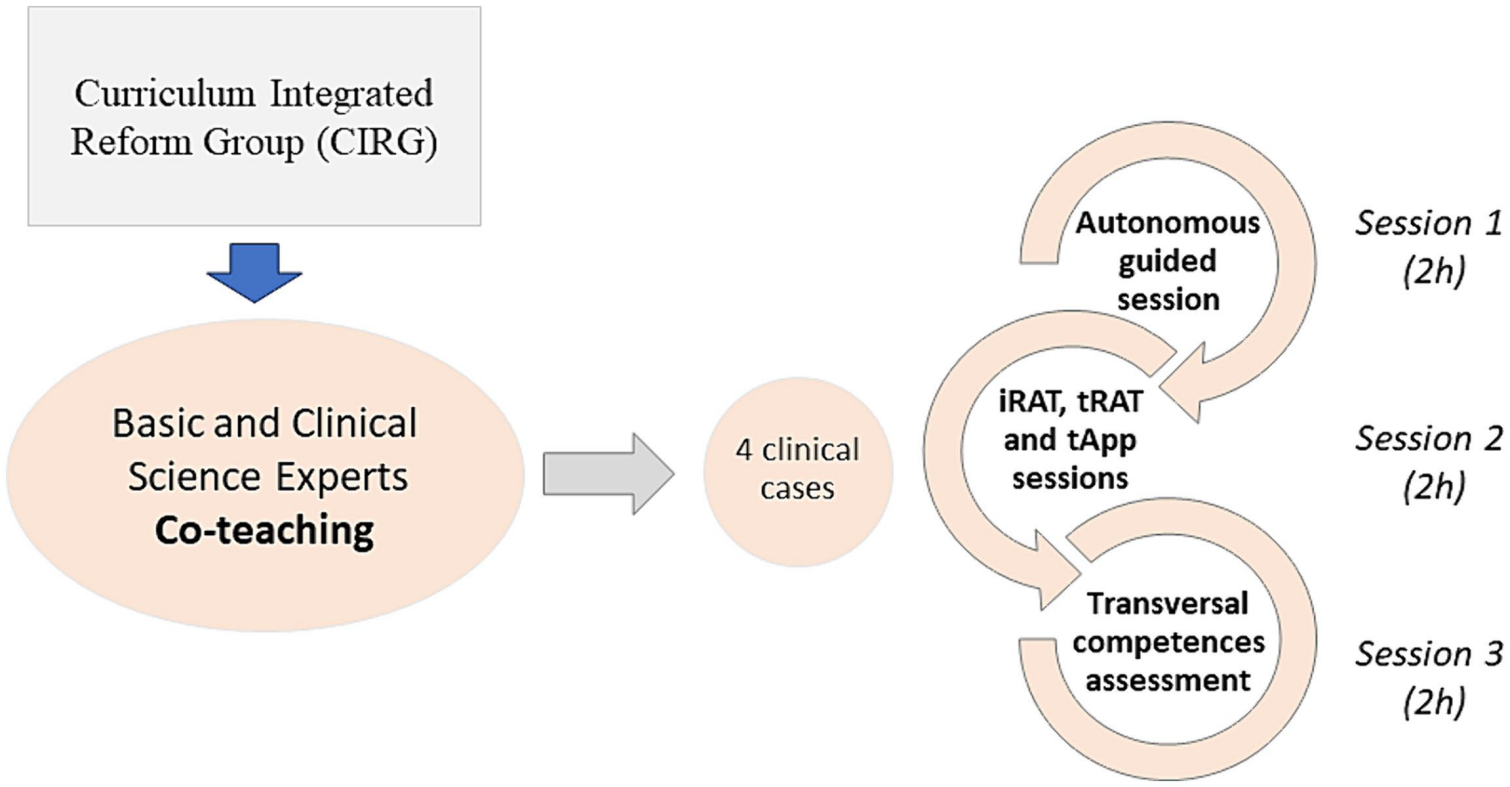
Schematic representation of the IM program.

Co-teaching was implemented throughout the whole IM program (Figure 1). For the first autonomous guided 2 h session, students used a limited and established material, prepared by the CIRG. They took advantage of the guidance and face-to-face collaboration and interaction between and with clinical and basic teachers in order to settle basic science principles and to relate them to clinical decision-making of real clinical scenarios (20). The TBL session emphasized basic science concepts relevant to the clinical case. The team application exercises (tApps) of the TBL were designed with different formats, including solving problems, working on reasoning questions and explaining clinical decisions around short case studies. Several clinical specialists contributed to tApp design to ensure relevance and accuracy of this second session. In the tApp every group of students made their own solutions of the exercise. Then, the solutions were discussed by the entire class, with the clinical and basic science instructors facilitating discussion and providing feedback and appropriate explanations. The last session focused on evaluating the two transversal competences mentioned before. These were evaluated both peer-to-peer (students assessing to each other by rubrics) and instructor-to-student (21).

Due to the situation generated by COVID-19, only the session on transversal competences that corresponded to clinical case 1 could be completed and evaluated in all groups.

### Data collection and analyses

For the qualitative study, teachers and students were asked to voluntarily participate in focus groups and semi-structured interviews. We conducted a focus group with three basic science teachers, two semi-structured interviews with clinical science’s teachers and one with a basic science teacher. Eight semi-structured students’ interviews were also conducted. The students were selected purposively according to their profile and motivation during the lessons, initial assessments, and academic performance. These were organized in 5 categories to cope all the profiles: high achiever, low achiever, leader, competent and poorly motivated. Both the interviews and the focus groups were recorded transcribed and consequently analyzed following a deductive approach (22, 23). The questions interviewers used in both cases to guide the conversation came from the theoretical framework that supports this work and the research objectives.

For the quantitative part, an analytical observational study of the 2019–2020 cohort of first-year undergraduate medical students was conducted (*n* = 220 students). The overall grades of the subjects included in the IM programme of the first-year medical students and the grade obtained by each of them in the IM programme were collected and coded to keep anonymity. The final grade of the IM program was calculated as the arithmetic media of the 4 real clinical cases. Each TBL session was evaluated as follows: second and third session equally weighted a 50%. Within the second session, the percentage of each part was 50% the iRAT, 25% the tRAT, and 25% the tApp.

A deductive thematic analysis was implemented to analyze the qualitative part. Initially all transcriptions are carefully read as well as informal notes taken by the interviewers after each interview or focus group. The second step consists of identifying the parts of the text that support or clarify the themes and thus begin to evolve into subthemes and categories. This process of working through and organizing the recorded experiences of both students and professors facilitates a better and more profound understanding of their opinions. This approach facilitated a structured framework for systematically and coherently interpreting participants’ responses (24). The thematic categories and their relationship to the interview questions are detailed in Table 1 for teachers and Table 2 for students.

**Table 1:** Themes, subthemes, codes and verbatim regarding teachers’ assessment of the Program.

Teachers			
Themes	Subthemes	Codes	Verbatim
Relationship between clinical and basic science teachers (1)	Enrichment of teachers’ own learning (1.1)	Learning (1.1.1)	BT.1 - *“The fact that we were basic and also clinical added even more, because in the specific subject I was able to learn things that I did not know from my colleagues who are clinicians”* BT.3 - “*The fact of working all together was very enriching”* CT.1 - “*From my clinical perspective, I realize that working with professors in the field of basic sciences broadens my range of knowledge and makes me have a completely different perspective from the one I usually have, and I think that enriches teaching towards the future student*” CT.2 - “(*it is beneficial….*) *to have a much more global vision than what clinicians can have, and to refresh the contents and to update ourselves in these matters that we are not used to*”
	It favors student learning (1.2)		CT.1 - “*the fact that teachers are able to learn while we are training our students, that enriches us a lot and I understand that in the long run the enrichment of the student has to be huge if this (program) is maintained over time, because it is like we are “feeding” ourselves*”
	Possible difficulties (1.3)	Clinical teachers availability (1.3.1)	BT.1 -"*As for practitioners, I was lucky because the two doctors we had, it is true that in some meetings …"I cannot because I’m on call…” but they always ended up doing their share and in addition, they were very good, they even knew colleagues to whom they asked questions… In other words, in our case they were very active, and they did participate*”
Knowledge integration (2)	Improving teacher learning (2.1)		CT.1 – “*It is necessary, because their perspectives are also different and that greatly improves patient care*” CT.2 - “*for me it was like rediscovering that to do the work that I do today, it is essential to have that (basic) knowledge*”
	Improvement of student learning (2.2)	By clinical integration (vertical) (2.2.1)	BT.1 - “*Bring the basic subjects closer to the clinical ones, and I think it also motivates them (students)*” BT.3 - *“they are motivated to see that later on they are learning things that they are going to see (in clinical contexts) and that they are going to have the criteria to know why something is happening”* CT.2 - *“when it comes to understanding, relating or linking some content to other, I think that it is the student’s overall vision that makes studying or learning things by relating… and I think it is much easier to learn by relating concepts than in isolation”*
		Integration (2.2.2)	BT.3 - “*I believe that what is offered to them by doing this type of activity is much greater than if each one of us works at these concepts (in class) separately*” BT.1 - “*It seems to me that the strong point is integrating subjects, that is, realizing that in the end knowledge is a whole that cannot be fragmented or made into pieces in your head*”
Methodology (3)	TBL structure (3.1)		BT.2 - “*I see it (TBL) as a good thing… Since everyone has to follow a fairly strict methodology, we do not end up doing our own thing … I think that’s what has enriched the project, that is, each one of us has not been able to fall into our individual teaching comfort zone, instead we were positively forced into the steps of the methodology, I think that this contributed to enriching each other*”
	Need of flexibility (3.2)		BT.1 - “*It is true that having to adapt to that methodology was like the base, but I missed a little more flexibility, or a little more freedom between the steps or perhaps focus some of the sessions in another way*”

BT, basic teacher; CT, clinical teacher.

**Table 2:** Themes, subthemes, codes and verbatim regarding students’ assessment of the Program.

Students			
Themes	Subthemes	Codes	Verbatim
Program’s impact on learning (4)	Motivation (4.1)	Professional practice (4.1.1)	S.2 – “*I think it’s a very good way of being reminded*: *“Hey, you are studying Medicine*” S.4 - “*It makes everything more dynamic, easier because you already find meaning in it, you see that it’s useful for something*”
		Methodology (4.1.2)	S.7 – “*Saying that it’s practical and it kind of changes our thinking (for the better) it’s that possibility of participation that motivates me”* S.6 - “*In general, with a practical approach, they tend to be a little more motivated*” S.2- “*We did not quite expect it to be so dynamic”*
	Engagement (4.2)	Readiness (4.2.1)	S.2 – *“It seems beneficial to me because in the end you will need to learn on your own throughout your career”* S.6 - *“(it helps to learn) complex content, yes, but I strongly believe that the student needs to work on it and acquire some understanding before the session and have previously made the information sort of their own”*
	Comprehension (4.3)		S.2 – *“For a student who struggles memorizing, if they see things in (situations which are similar to) real life, it is much easier”* S.8 - *“It helped us a little to understand it, bring what we have studied to real life, to cases that could really happen”* S.5 - *“These activities facilitate the understanding of complex content”*
Relationships (5)	Professor-Professor (5.1)	Importance of collaboration (5.1.1)	S.4 – *“It was great to have two professors in the same session”* S.1 - *“The good thing is that since there was more than one professor, no group of students felt isolated “* S.3 - *“I did notice a different atmosphere because I know it was a personal project of our professors who were collaborating… I felt that it added a special charm to also engage the student, and I honestly have enjoyed better (than other more conventional classes), I personally liked it”* S.2 - *“In the end you see professors of different subjects collaborate and you see that what you are working on in a particular course with one professor has to do with what the other professor also explained to you but in a different subject, I think it’s a really good idea”*
	Professor-student (5.2)	Added value (5.2.1)	S.8 – *“It was a much closer (interaction) than in other types of practical work”* S.5 - *“Maybe in Integrated Medicine there was more interaction… it’s different because in the end it’s like some sort of closeness (with professors) and a little more conversation, not just: “I have a question and he/she answers it”*
Integration (6)	Application (6.1)		S.6 - *“I found it interesting that from the very first year the basic subjects such as Biochemistry, Genetics, Physiology, Anatomy, can be related to future clinical cases that as doctors you encounter in daily clinical practice. It is very interesting that first-year students see that everything they are studying has a meaning, a purpose and an applicability”* S.1 *- “Professors in the program tried to relate what we saw in lectures, which is more theoretical, more to memorize, and understand, somewhat abstract concepts, and associate them with clinical reality”* S.8 *- “I think it really helps to understand and integrate beyond what you can study (on your own)”* S.5 *- “It is very good to see that the things that are studied in the end can also be applied to real clinical cases”*
	Horizontal integration (6.2)		S.5 - “*Personally, it was quite good for me to see that the things that were studied in one course could be complemented with those of other subjects*”
	Vertical integration (6.3)	Professional practice (6.3.1)	S.8 - “*Seeing clinical cases, investigate a little what happens to each patient research, investigate each case, it brings you closer or encourages you a little more (to what Medicine is)”*
Team work (7)	Benefitial (7.1)		S.5 – “(*Working in a group*) *in the end everyone contributes something and you also learn from your peers and what they know*”
	Difficulties (7.2)		S.3 - *Well, team-based learning is fine for people who are really interested, but if you get into a group where people are just not interested, they do not come prepared and they do not really want to participate, deep down it can be quite frustrating, It does not make me angry, but it’s a bit disappointing when it comes to learning and you realize: “I’m alone in the group*”“
Integrated medicine within the curriculum (8)	Continuity (8.1)		S.1 – “*This implementation (of the program) would be something positive throughout the entire degree and I think it could be something significant that will help later in our profession*”
	Excesive burden (8.2)		S.7 - “*If it is organized like an extra course, something positive could be perceived as negative.* “

S, student.

To evaluate the students’ perception of the IM program, a modified survey from the-National Student Survey (thestudentsurvey.com) was conducted. A total of 107 students completed the survey. To analyze the descriptive study, responses to questions were grouped into 5 categories: professors as facilitators, student experience with IM program, organization and resources of IM program, student collaboration, and assessment.

**Figure 2:**
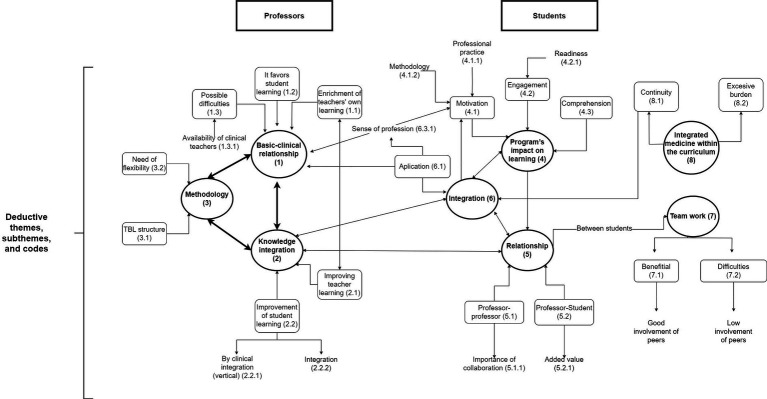
Results from relational analysis.

Results from relational analyses are summarized in Figure 2, which establishes the links between the different themes, subthemes and codes reported before. This figure identifies the main logical relations within and between students and professors. As can be seen, there is substantial interaction between perceptions from students and teachers, supporting the value of the co-teaching experience in basic and clinical sciences.

For qualitative analysis, we looked for code-code (and ultimately) verbatim logical relationships based on meaning from the different themes, subthemes and codes (23). Results from the relational analysis were conceptualized as linking arrows between the different themes and subthemes manually using diagrams.net.

## Results

In this section, we present results from the qualitative and the quantitative approaches together to provide a thorough assessment of the teachers’ and students’ perspectives regarding SCT. In Tables 1, 2, we summarize the analysis of themes and subthemes, as well as we provide examples of the supporting verbatim. To facilitate understanding, themes and subthemes are identified with a number and sub-numbers (in parentheses) ranging from 1 to 3 for teachers (Table 1) and from 4 to 8 for students (Table 2). In the following subsections we will be describing results of students and teachers together for a particular theme, and so the numerical codes will be intermixed. Quantitative results are reported in Table 3.

**Table 3:** Descriptive statistical analysis of results from the questionaries in percentages.

Categories	Item number	Number of answers	Likert (%)				
			1	2	3	4	5
Professors as facilitators	8	852	3.1	3.7	11.6	27.3	54.2
Student experience with IM program	5	532	3.9	6.2	14.1	28.4	47.4
Organization and resources of IM program	6	640	6.4	8.4	16.1	24.8	44.2
Student collaboration	3	321	3.7	4.0	11.2	25.5	55.4
Assessment	2	232	2.3	2.3	11.3	22.1	61.8

### Relationship between clinical and basic science teachers

One of the themes that teachers highlighted with great importance was the relationship between basic science and clinical teachers (Table 1, Theme 1). All of them were specially satisfied with this collaboration, which they considered as a rewarding experience that permitted them to increase their knowledge (subtheme 1.1). This aspect is particularly interesting, since it shows the need to work collaboratively amongst professors of both traditions. For example, according to a clinical teacher *“From my clinical perspective I realize that working with professors in the field of basic sciences broadens my range of knowledge …”* (Table 1, CT.1), while a basic teacher emphasized the fact that *“…was able to learn things that … did not know from colleagues who are clinicians”* (BT.1). On the other hand, in this basic-clinical relationship, they perceived that shared teaching is a determining factor that favors student learning (subtheme 1.2). Importantly, they also pointed out the difficulties that educators have faced with this approach (subtheme 1.3). These were especially due to the workload of clinical teachers (Table 1, code 1.3.1), which compromises their availability and sometimes makes it difficult to organize meetings for joint planning.

Along these lines, students positively valued the close relationship between professors (Table 2, subtheme 5.1). They particularly appreciated that *“…you see professors of different subjects collaborate…”* (code 5.1.1, S.2) and the strong relationship between professors and students that this program offered (subtheme 5.2). In this sense, they highlighted the advantage of having a closer interaction to favor availability of teachers *“…than in other type of practical work”* (code 5.2.1, S.8).

Similar perceptions were found in the quantitative analysis of the survey, where 81.6% of the students expressed a positive rating of the teaching faculty (scoring 4 or 5; Table 3). This is consistent with students highlighting aspects such as their availability to solve questions and to make them feeling part of the academic community. Similar percentage of students (81.6%) valued the interaction with their classmates very positively too (with 4 or 5; Table 3). Therefore, SCT in the IM program was considered an overall positive experience by both teachers and students.

### Knowledge integration

Regarding knowledge integration between the basic and clinical content (Table 1, theme 2), the interviewed teachers emphasized the importance of the relationship between faculty (Table 1, theme 2). They saw this interaction *“…as necessary, because their perspectives are also different…”* (CT.1). According to them, this integration leads to an improvement in their own learning (subtheme 2.1). First, they stated that integration avoids a common fragmented approach to knowledge (code 2.2.1, CT.2). Secondly, they highlighted that this integration is essential to improve student learning (subtheme 2.2) both when working on clinical concepts (code 2.2.1, BT.1, BT.3 and CT.2) and in general (code 2.2.2, BT.1 and BT.3).

Students also valued integration, emphasizing its meaning and applicability (Table 2, theme 6). They *“…found it interesting that from the very first year the basic subjects such as Biochemistry, Genetics, Physiology, Anatomy, can be related to future clinical cases that as doctors you encounter in daily clinical practice…”* (subtheme 6.1, S.6). Students mentioned the benefits of horizontal integration (subtheme 6.2) in terms of the relationship between the basic subjects. They also referred to vertical integration (subtheme 6.3), which was specifically related to professional practice that permitted them *“…investigate a little what happens to each patient*…*”* (code 6.3.1; S.8). This supported the applicability of basic knowledge to future clinical practice from the beginning of their training.

### Methodology

Another topic of interest was the methodology used in the program (Table 1, theme 3). Interviewed professors highlighted that the application of this methodology allows the sessions to be structured more effectively. For example, *“I see it (TBL) as a good thing… Since everyone has to follow a fairly strict methodology, we do not end up doing our own thing…”* (subtheme 3.1, BT.1). However, they also suggested that it might work well with some degree of flexibility (subtheme 3.2).

Students perceived that the methodology promoted motivation (Table 2, subtheme 4.1) by providing a significant connection with professional experience, making *“… everything more dynamic, easier because you already find meaning…”* (code 4.1.1, BT.1). They found this experience closer to future professional practice (subtheme 6.1), which may favor understanding of clinical concepts (code 4.1.2). Possibly, these evaluations (Table 2, theme 4) are influenced by the structure of the IM program, and in this sense the students affirmed that the program requires commitment (subtheme 4.2) and preparation which *“… seems beneficial … to learn on your own…”* (code 4.2.1, S.2). Overall, students perceived an improvement in comprehension and retention of the content (subtheme 4.3).

### Team work

Among the aspects that we sought to investigate, the perception of teamwork was especially considered (Table 2, theme 7). In general, students reflected that the methodology has a beneficial effect because *“… working in a group in the end everyone contributes … and you also learn from your peers…”* (subtheme 7.1, S.5). Importantly, they also mentioned the importance of commitment and that everyone in the team must be involved (subtheme 7.2). This was identified as a drawback in some cases.

### Integrated medicine and curricular design

Finally, in relation to the general perception of students regarding the inclusion of the IM program in the curriculum (Table 2, theme 8), two relevant subthemes were found. On the one hand, students considered that this program presented a great opportunity and expressed their interest in maintaining it in the future (subtheme 8.1). This qualitative data is supported by the quantitative analysis of the surveys, since 75.8% were satisfied and very satisfied with the program (Table 3).

However, some students perceived the program as of an excessive workload, *“… organized like an extra course…”* (subtheme 8.2, S.7). This perception had echoes in the survey, where the lowest score pertained to the timing and scheduling of IM, out of the standard timetable. This was interpreted by students as an additional burden.

### Relational analysis

Results from relational analyses are summarized in Figure 2, which establishes the links between the different themes and subthemes reported before. This figure identifies the main theme-theme relations within and between students and professors. As can be seen, there is substantial interaction between perceptions from students and teachers, supporting the value of the co-teaching experience in basic and clinical sciences.

## Discussion

With this work, we aimed to investigate the students’ and teachers’ perception of co-teaching as a pedagogical approach to integrate basic and clinical sciences. Co-teaching involves two or more teachers collaborating to plan, deliver, and evaluate together their own contents, thus creating a dynamic learning environment. In our case, the focus is on integrating basic sciences (such as anatomy, physiology, and biochemistry) with their clinical application. Traditionally, these subjects have been taught separately, leading to a perceived disconnection between theoretical knowledge and its practical application. By exploring professor and students’ perceptions of co-teaching, we gained insights into whether this approach effectively bridges the gap between basic and clinical sciences.

Our qualitative and quantitative results demonstrate that teachers value co-teaching as a determining tool that favors student learning. This is depicted in Figure 2, where the comments on Basic-clinical relationship (Theme 1) where found in close interaction with those for Integration (2) for professors. In fact, it has been described that co-teaching could be considered as a substitute for an integrated curriculum in medical education (25). Clinical teachers state that this approach gives the students an overall vision that helps in integrating basic and clinical concepts. The students perceive that our IM program makes their learning experience better. They value very positively having a closer interaction with basic and clinical teachers. This is shown as the theme 5 (Relationship) for students in close interaction with Integration for both professors (theme 2) and students (theme 6) in Figure 2. This result is consistent with conclusions from a systematic review on the use of SCT in medical sciences (26). In that study, students stated that they were more engaged in the learning process, and that their learning experience was optimized in a course directed by SCT (26). Our students feel particularly positive about teachers of different subjects working together, supporting the importance of vertical integration and synergy (Figure 2; Relationship, theme 5). A significant majority of our students report that they understood better the connection between basic and clinical sciences when contents were co-taught (Figure 2, see interaction between Integration themes 2 and 6). These positive perceptions were reflected in better exam outcomes for content covered in co-taught over solo-taught sessions as it has been observed in other studies (27).

Importantly, consistent with other studies, we also identified concerns and drawbacks of SCT which are worth mentioning. In particular, some mismatch and lack of coordination between teachers were highlighted, and this was considered confusing and distracting for the class (26). This emphasizes the importance of careful planning, collaboration, and commitment among faculty to implement SCT (27). Being aware of this limitation, we created a curriculum integration reform group (CIRG), which provided support, TBL formative actions and careful planning of the IM program.

Our students also perceived that co-teaching made them learn actively from immediate feedback they could get from the basic and clinical teachers sharing the same learning environment. With multiple teachers in the classroom, they have more opportunities for interaction, discussions, and hands-on activities. This collaborative environment can foster critical thinking, problem-solving, and teamwork skills, which are essential for healthcare professionals. Additionally, co-teaching promotes motivation and active student engagement and participation (28). This is depicted in Figure 2 as connections between theme 4 (Perception) and 6 (Integration) for students, and particularly between motivation (4.1) in close interaction with the theme 1 (Basic-clinical relationship) of professors. Working in teams fosters collaboration, communication, and peer-to-peer learning. In fact, our students agree that teamwork and student’s interaction are beneficial for learning. Importantly, this comes with a limitation since clinical teachers are typically busy with their clinical assistance duties, something that should be carefully considered in the deployment of this sort of programs (Figure 2, Team work theme 7 for students).

Horizontal integration of basic sciences, such as Anatomy, Physiology, and Biochemistry, within a TBL approach offers several potential benefits for an IM program (Figure 2, theme 3, Methodology for teachers and Theme 8 MI program for students). TBL is a collaborative learning strategy that involves students working in teams to solve problems and apply their knowledge to real-life scenarios. One advantage of using TBL in the integration of basic sciences is that it promotes student engagement (29). In TBL sessions, students work in small groups to solve clinical case scenarios, applying their knowledge of basic sciences to diagnose and manage patients. This active participation allows students to actively apply their theoretical knowledge, enhancing their critical thinking and problem-solving skills (30).

The use of real clinical case scenarios adds authenticity and relevance to the learning experience and to the IM program specifically. By presenting students with realistic patient cases, they are exposed to the complexity and variability of clinical practice. This integration also reinforces the practical application of theoretical concepts, as students can directly observe how their knowledge translates into patient care.

It is important to consider potential challenges in implementing this objective. Integrating basic sciences within a TBL framework requires careful curriculum design and coordination between basic science and clinical faculty. Faculty development programs may be necessary to ensure instructors have the necessary skills to facilitate TBL sessions effectively (31). Additionally, logistics, such as the availability of appropriate case scenarios and resources, need to be considered.

## Conclusion

Our work supports the value of co-teaching in basic and clinical sciences within a TBL framework set in real clinical case scenarios. By employing this approach, students can actively apply their theoretical knowledge to clinical practice, enhancing their critical thinking, problem-solving, and clinical reasoning skills. By investigating the benefits and limitations of co-teaching, we can gain insights into its effectiveness in bridging the gap between theoretical knowledge and practical application. The findings of this study can inform curriculum design and improved educational practice, leading to enhanced learning experiences for healthcare students and ultimately better patient care.

## Data availability statement

The raw data supporting the conclusions of this article will be made available by the authors, without undue reservation.

## Author contributions

IR-M: Investigation, Project administration, Writing – original draft, Writing – review & editing, Methodology, Resources. EC: Investigation, Methodology, Writing – original draft, Writing – review & editing, Conceptualization. JS-G: Investigation, Methodology, Writing – original draft, Writing – review & editing, Data curation, Formal analysis. CA: Data curation, Formal analysis, Investigation, Writing – original draft, Writing – review & editing. AST: Investigation, Writing – original draft, Writing – review & editing, Methodology, Project administration, Resources. ML: Methodology, Writing – original draft, Writing – review & editing, Formal analysis, Validation. BG: Writing – original draft, Writing – review & editing, Conceptualization, Data curation, Funding acquisition, Investigation, Project administration, Supervision.
